# Comparison of smokers’ mortality with non-smokers following out-of-hospital cardiac arrests: a systematic review and meta-analysis

**DOI:** 10.1186/s41043-024-00510-w

**Published:** 2024-04-26

**Authors:** Nai Zhang, Yu-Juan Liu, Chuang Yang, Peng Zeng, Tao Gong, Lu Tao, Ying Zheng, Shuang-Hu Dong

**Affiliations:** 1 Department of Emergency, Jiangxi Province Hospital of Integrated Chinese and Western Medicine, 90 Bayi Avenue, Nanchang, 330003 China; 2 Department of Intensive Care Unit, Jiangxi Province Hospital of Integrated Chinese and Western Medicine, Nanchang, 330003 China

**Keywords:** Smoking, Mortality, Out of hospital, Cardiac arrest, Meta-analysis

## Abstract

**Objective:**

Although some studies have linked smoking to mortality after out-of-hospital cardiac arrests (OHCAs), data regarding smoking and mortality after OHCAs have not yet been discussed in a meta-analysis. Thus, this study conducted this systematic review to clarify the association.

**Methods:**

The study searched Medline-PubMed, Web of Science, Embase and Cochrane libraries between January 1972 and July 2022 for studies that evaluated the association between smoking and mortality after OHCAs. Studies that reportedly showed relative risk estimates with 95% confidence intervals (CIs) were included.

**Results:**

Incorporating a collective of five studies comprising 2477 participants, the analysis revealed a lower mortality risk among smokers in the aftermath of OHCAs compared with non-smokers (odds ratio: 0.77; 95% CI 0.61–0.96; *P* < 0.05). Egger's test showed no publication bias in the relationship between smoking and mortality after OHCAs.

**Conclusions:**

After experiencing OHCAs, smokers had lower mortality than non-smokers. However, due to the lack of data, this ‘smoker’s paradox’ still needs other covariate effects and further studies to be considered valid.

## Introduction

Out-of-hospital cardiac arrests (OHCAs) are a major challenge to global health and have high morbidity and mortality rates [1–3]. More than 345,000 OHCAs (1.4% incidence) are reported annually in America [4]. In England, about 80,000 people per year have an OHCA [5]. Survival rates following OHCAs in Europe are notably limited, ranging between 0.6 and 25% [1, 6]. In stark contrast, OHCA incidents represent less than 1% of reported cases in China [7]. It is noteworthy to mention that coronavirus disease (COVID-19) reportedly increases mortality rates for OHCAs, as severe acute respiratory syndrome coronavirus 2 (SARS-CoV-2) interacts closely with angiotensin-converting enzyme-2 (ACE2) receptors on myocardial cells that could mediate SARS-CoV-2 into myocardial cells and cause cardiotoxicity [8, 9].

Smoking is a major public health challenge affecting human health worldwide [10], and approximately 7 million deaths are attributed to smoking each year [11]. The risk of premature mortality is three times higher for smokers than non-smokers [12]. Smoking significantly influences morbidity and mortality of various diseases, including cardiovascular diseases [12]. These also place a significant financial burden not only on the national healthcare systems but also on individuals, which has an upward trend every year [13–15].

Several factors are associated with the mortality of patients with OHCA, such as obesity [16], diabetes [17], age [18], smoking, coronary heart disease [19], cardiopulmonary resuscitation time [20] and quality of cardiopulmonary resuscitation [20]. However, the relationship between smoking and mortality following OHCAs remains unclear and needs further investigation. While recent studies have demonstrated conflicting findings, some studies have shown that smoking is strongly associated with an increased risk for cardiovascular disease that increases the incidence and mortality rate following OHCAs [21, 22]. Lahmann et al. [23] demonstrated conflicting findings, as smoking lowered the mortality rate and improved the prognosis of neurological functions, termed the ‘smoker's paradox’.

Due to the conflicting findings, the purpose of this study was to perform a comprehensive assessment of the impact of smoking on patient prognosis after OHCAs using existing literature evidence. A meta-analysis and systematic review will determine the relationship between smoking and mortality-related outcomes in patients with OHCAs. Finally, the study will provide a theoretical basis for resolving the ‘smoker's paradox’.

## Methods

### Ethical considerations

Previously published studies, which described prospective and retrospective studies and did not need ethical approval, were included.

### Search strategy

This review was registered in the International Prospective Register of Systematic Reviews (PROSPERO) (CRD42022361239). This study was conducted following Preferred Reporting Items for Systematic Reviews and Meta-Analysis guidelines. Literature searches on the Medline-PubMed, Web of Science, Embase and Cochrane libraries for trial studies between January 1972 and July 2022 were included. To ensure data integrity, the study was also combined with manual research. Keywords and related terms were used, such as ‘cardiac arrest’, ‘heart arrest’, ‘cardiopulmonary arrest’, ‘smoking’, and ‘behaviors, smoking’. There were no language restrictions on the publication of this study.

### Selection criteria

Articles were included if the study evaluated mortality in smokers with cardiac arrest through odds ratios (OR), hazard ratios (HR), or relative risk (RR) ratios with 95% confidence intervals (CIs). All patients were older than 18 years of age. This analysis included case–control or cohort studies comparing exposure factors between both groups. Non-smokers with OHCA were used as a control group.

Patients who suffered from a drug overdose, trauma, asphyxiation or electrocution due to cardiac arrest were excluded, along with their respective reviews. Additionally, there was an assessment of the literature's quality, and any studies characterised by low-quality, incomplete datasets or those in which the data could not be ascertained from the literature due to insufficient descriptions were systematically excluded.

### Data extraction

Two reviewers independently collected titles, first author names, research methods, smoking events, smokers, mortalities, abstracts and full texts of the obtained reviews. Studies were screened according to their admission and exclusion criteria, and all relevant data based on the pre-designed form were extracted. After screening, the data were cross-checked. When the opinions of the authors were inconsistent, they were resolved through discussion or consultation with a third person and were finally included in the study.

### Quality assessment

The Newcastle–Ottawa scale (NOS) scoring system [24] was used to evaluate the methodological quality of included studies, and the principle of the star system was adopted. The NOS scoring system comprises three main parts, with a maximum of nine stars.

### Statistical analysis

STATA 16.1 was used to test the heterogeneity of collected information and synthesise the summary RR, OR and HR values with their corresponding 95% CIs depending on the type of study. The χ^2^ and *I*-square (I2) tests were used to test the heterogeneity of the included studies. When *P* was > 0.05 and *I*^*2*^ was < 50%, there was no statistical heterogeneity among the studies, and the fixed effect model was used to analyse the study. When *P* was < 0.05 and *I*^*2*^ was > 50%, heterogeneity was considered significant among the studies, and the source of heterogeneity was further searched. If heterogeneity could not be eliminated and clinical consistency was found, the random effects model and subgroup analysis were used for statistical literature analysis; otherwise, descriptive analysis was used. Potential publication bias was analysed by Egger's analysis and funnel plots. Two reviewers autonomously evaluated the risk of bias within individual studies employing the Cochrane Risk of Bias Tool for Randomised Trials, version 2. In cases of dissenting assessments, consensus was achieved through dialogue. The evaluation of bias risk was conducted for each outcome encompassed within the trial. Yet, it was reported at the trial level, encapsulating the highest risk of bias score across all outcomes. The stability of meta-analysis results was tested by sensitivity analysis: Documents were excluded one by one. Each included study was eliminated one by one before merging effect sizes. The inclusion and exclusion criteria were changed, or certain types of literature were excluded before merging effect sizes.

## Results

### Search results

**Fig. 1:**
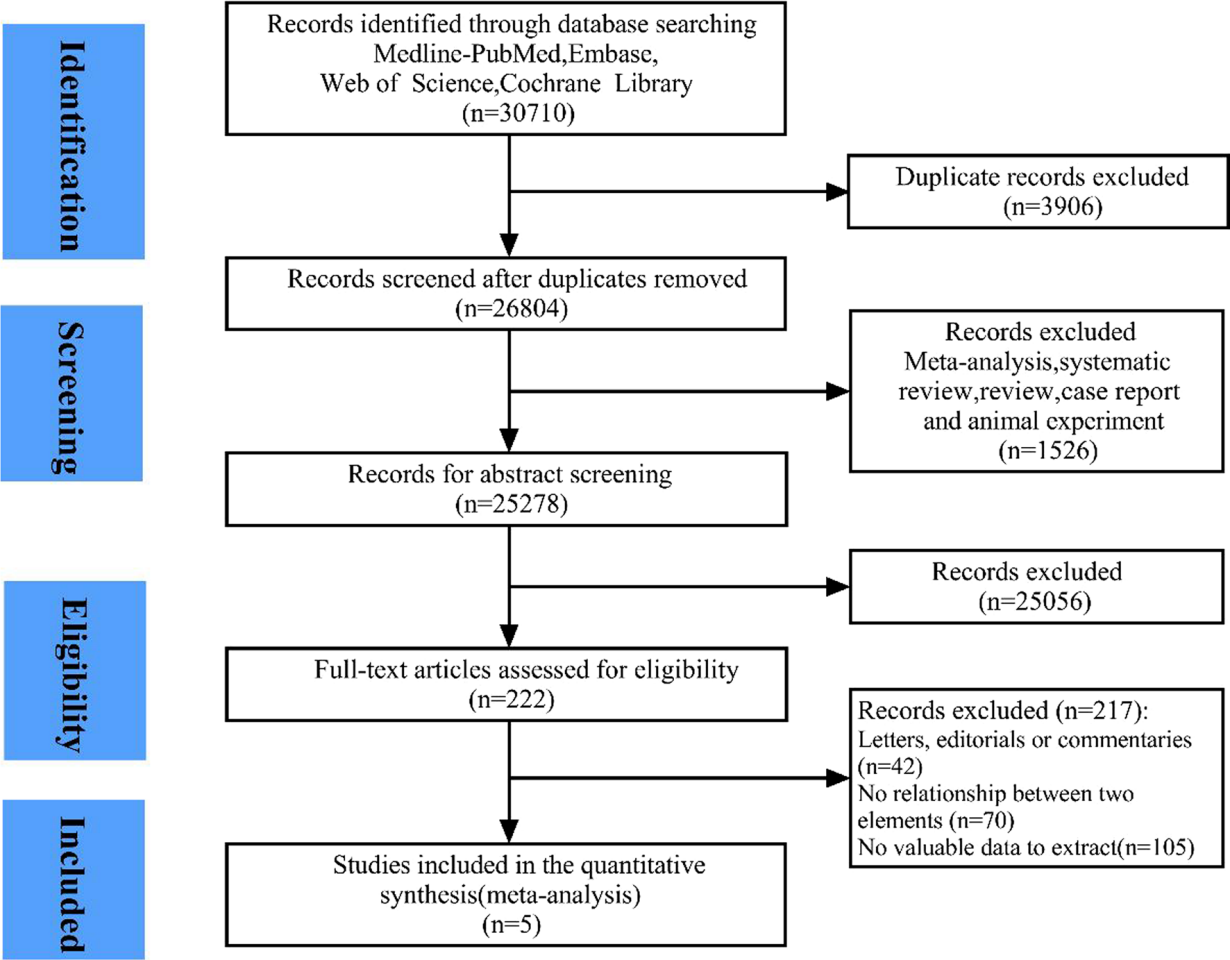
Flowchart of the study

The study’s search identified 30,710 relevant citations, and after removing duplicates, 26,804 studies were screened. A total of 1,526 studies were excluded, as they were animal experiments, reviews and case reports. After reading the abstract, 25,056 studies were excluded. Thus, 222 studies were evaluated with the full text in detail, and 217 were excluded. Among them, 42 were letters, editorials or commentaries, and 70 showed no relationship between smoking and mortality caused by cardiac arrest. The remaining 105 studies did not have a relevant date. Finally, only five studies [25–29] were included in this meta-analysis (Fig. 1). Three studies had an NOS score of 6, and the others had NOS scores of 7 and 8. All studies had NOS scores over 6 (Table 1).

**Table 1:** Characteristics of the studies included in the meta-analysis

Author/year	Study type	Country	Cohort size	Follow-up duration	Recruitment time	Selection	Comparability	Outcome	NOS score
Albizreh [25] 2021	Retrospective	Qatar	1144	Until hospital discharge	1991–2013	★★	★★	★★	6
Pollock [26] 2014	Prospective	United States	181	Until hospital discharge	May 2007 and January 2012	★★★	★★	★★★	8
Arrich [27] 2006	Retrospective	Austria	774	Until hospital discharge	September 1991 and December 2004	★★★	★★	★★	7
Leick [28] 2013	Retrospective	German	28	30 days	January 2010 and December 2011	★★	★★	★★	6
Whittaker [29] 2016	Retrospective	United States	350	Until hospital discharge	2008–2013	★★	★★	★★	6

Each star (★) represents one point in the NOS score

*NOS* Newcastle–Ottawa scale

### Characteristics of selected studies

Five studies were included in this meta-analysis, and a total of 2903 participants were involved. Four studies were retrospective, and one was a prospective study. All included studies were published between 2006 and 2021 (Table 2).

**Table 2:** Baseline data of the studies included in the meta-analysis

Author/year	Sample size	Group (Age, years)		Male (%)	Smokers (%)	Diabetes (%)	Hypertension (%)	STEMI (%)	Mortality (%)
Albizreh [25] 2021	1146	OHCA with age ≤ 40 years	(*n* = 159; 13.9%)	73.3	20.8	43.3	44.1	15.4	83.6
		OHAC with age > 40 years	(*n* = 985; 86.1%)						
Pollock [26] 2014	188	Non-smokers	62 (49–73);	64.1	52.5	NA	NA	22.1	60
		Smokers	59 (52–64)						
Arrich [27] 2006	1191	In-Hospital Mortality	62 ± 15	73	32.7	16	28	NA	56.2
		Unfavorable Outcome	60 ± 13						
Leick [28] 2013	28	Death	53.9 ± 12.9	53.6	32.1	28.6	71.4	42.9	60.7
		Alive	60.3 ± 9.6						
Whittaker [29] 2016	350	Alive	60.6 ± 13.1	68	20	12	42	56	67.4
		Death	69.0 ± 17.2						

*STEMI* ST-elevation myocardial infarction

### Meta-analysis for mortality

**Fig. 2:**
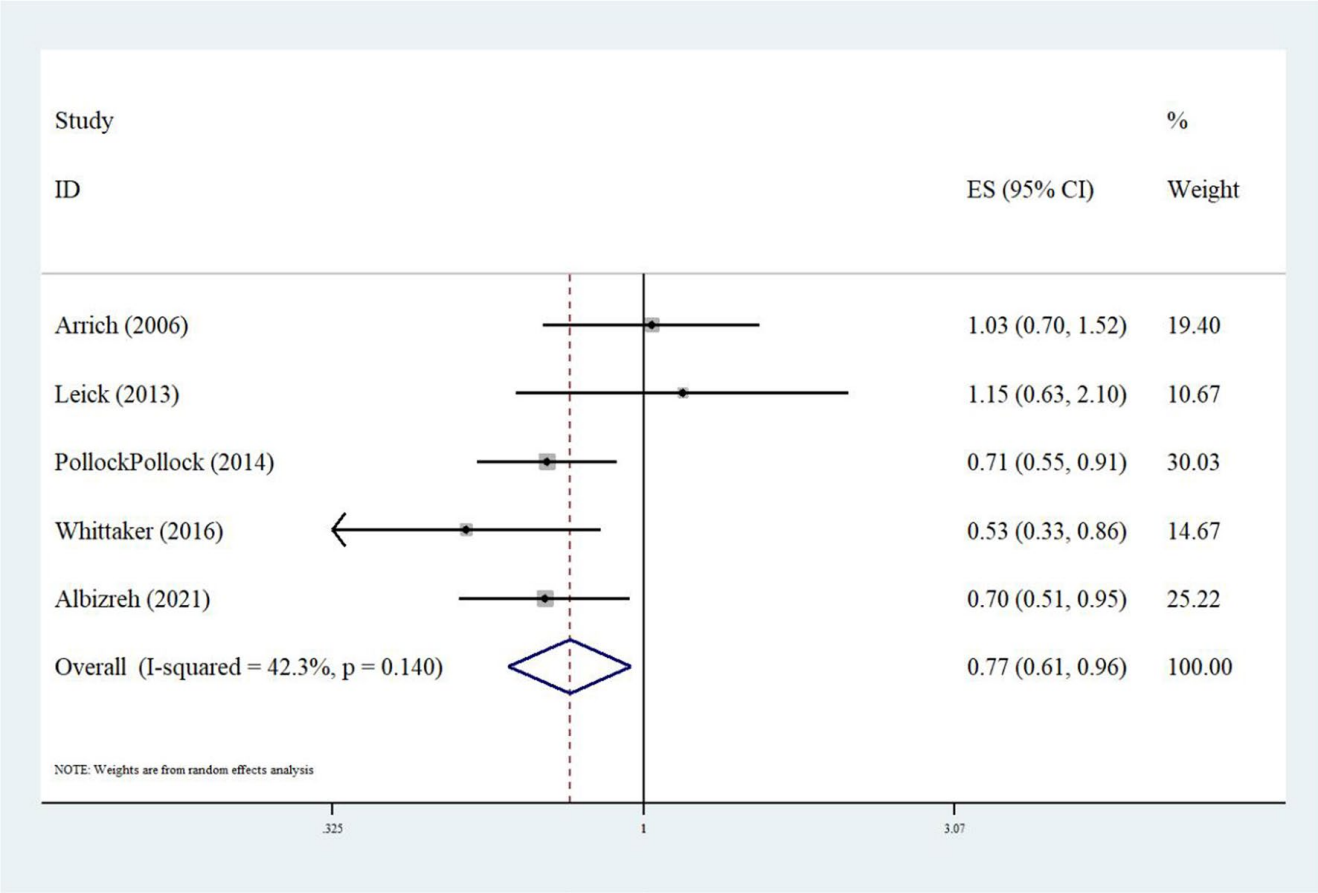
Forest plot regarding the risk of death between smokers vs. non-smokers with out-of-hospital cardiac arrest

A heterogeneity test was performed in this study and showed mild heterogeneity. (*I*^*2*^ = 42.3%; *P* = 0.140 > 0.1) (Fig. 2). The forest plot found that smokers have lower mortality compared to non-smokers (HR: 0.77; 95% CI: 0.61–0.96), and the overall effect was 2.37 (*P* = 0.018 < 0.05), showing a statistically significant difference between the groups.

### Sensitivity analysis and funnel plot analysis

**Fig. 3:**
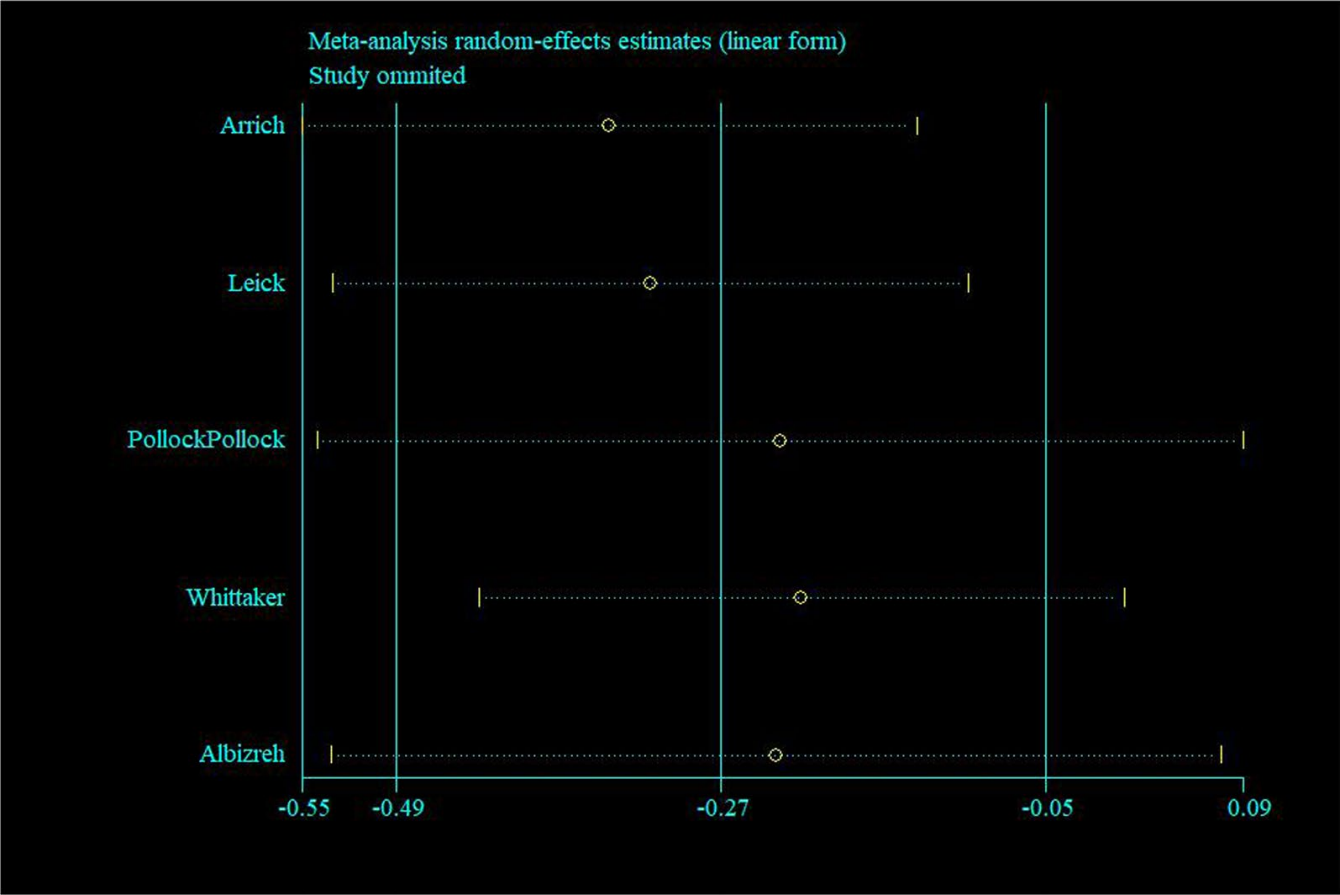
Sensitive analysis regarding the risk of death between smokers and non-smokers with out-of-hospital cardiac arrest

**Fig. 4:**
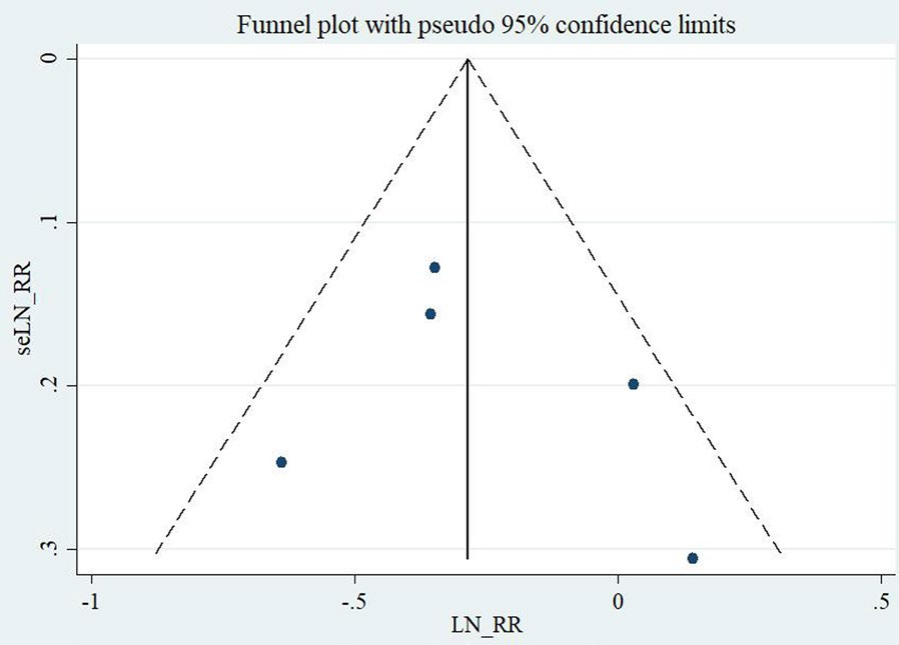
Funnel plot of the included publications

Sensitivity analysis regarding mortality risk indicated that results did not significantly change due to the impact on each study (Fig. 3). Funnel plots indicated symmetrical distribution in this study, which suggested no significant evidence of bias in all included publications (Fig. 4). Egger’s regression asymmetry tests showed no significant bias among the five studies (*P* = 0.65 > 0.05).

## Discussion

This meta-analysis systematically evaluated the latest evidence regarding the comparison of mortality between smokers and non-smokers after they experienced OHCAs. Upon the inclusion of 2477 patients from a compilation of five studies, this study revealed a notably diminished mortality risk among smokers compared to non-smokers following OHCAs. These findings allude to the presence of a phenomenon commonly referred to as the ‘smoker’s paradox’. However, this phenomenon still awaits validation in subsequent large, high-quality studies.

Patients with OHCAs have high mortality rates and experience considerable healthcare costs. Many studies explored the factors that influenced the prognosis of OHCAs. For instance, previous meta-analyses demonstrated that women experienced slight differences among those who survived OHCAs and were discharged [30]. Patients who underwent cardiopulmonary resuscitation before arriving at the hospital had an increased 30-day survival after OHCAs [20]. Previous studies may have suggested a potential association between smoking and unfavourable outcomes regarding OHCA-related mortality. No single epidemiological meta-analysis contradicts the increased risk of smoking regarding mortality from OHCA. Smoking is well known to be bad for public health, whereas this study’s results showed smokers had lower mortality rates than non-smokers, thus named the ‘smoker's paradox’.

The smoker's paradox was recognized a few decades ago in patients with acute myocardial infarction [31], and its existence has been continuously reported in subsequent studies. In 2017, data derived from the 2013–2016 China Acute Myocardial Infarction Registration study, encompassing 40,640 patients diagnosed with acute myocardial infarction and conducted by Fuwai Hospital, unveiled a noteworthy observation. The findings indicated that smoking persisted as a mitigating factor in in-hospital mortality [32]. A recent study found that a patient’s current smoking status is related to lower susceptibility to COVID-19 infection [33]. A pooled analysis of 10 randomised studies published in the Journal of the American College of Cardiology in 2020 of patients with ST-elevation myocardial infarction undergoing coronary intervention found that smokers had lower mortality rates within 1 year than non-smokers. Smokers had higher mortality risk after adjustments for age and other risk factors, and the results suggested short-term prognosis was better in smoking patients who suffered heart attacks simply because smokers were younger and had fewer cardiovascular risk factors [34].

However, the independent association between smoking and favourable outcomes after cardiac arrest remains unknown. It is understood that smoking may protect against ischemic injury, as the lack of oxygen caused by smoking may produce an ischemic precondition that could reduce the mortality of ischaemia–reperfusion injury. Ischemic conditioning was originally described by Murry et al. [35]. Hausenoly et al. [36] discovered that when transient nonfatal ischaemia and reperfusion were applied to the organ or tissue bed, it could prevent future reperfusion injury. Recent clinical studies showed that ischemic conditioning was beneficial in alleviating ischemic reperfusion [37–39]. Reperfusion is essential in maintaining vital signs and survival. In addition, studies suggested that acute myocardial infarction was the leading cause of cardiac arrest in patients. Smoking can reduce the infarction size by decreasing ischaemia reperfusions [40]. A new HUNT study from Norway discovered that both current and former smokers had lower levels of high-sensitivity cardiac troponin I (hs-cTnI) than non-smokers, and there existed a dose–response relationship between hs-cTnI and smoking in past smokers. It could be possible that the composition of tobacco could induce ischaemia preconditioning, which would make the heart muscle more resistant to ischemic damage and have a protective effect on the heart. This was found in smokers, which could partly explain the ‘smoker’s paradox’ [41].

### Strengths and limitations

This meta-analysis has some limitations that must be acknowledged. First, most of the results were based on unadjusted estimates; therefore, future studies should evaluate the impact of other known confounding variables, such as gender, age, body mass index and lifestyle, on the risk of mortality from OHCA among smokers. Second, despite the low heterogeneity found across publications, the study cannot rule out the possibility that other inadequately measured factors may bias the associations. Third, former smokers, current smokers, packs of cigarettes per day and total years of smoking were not reported in this study, so it was impossible to perform a detailed differentiation between patients. Therefore, future studies that research mortality from smoking and OHCA are needed.

However, there are many advantages to this study. Two researchers independently assessed the quality of the included studies using the NOS, and publication bias was evaluated by a funnel plot analysis together with Egger’s test, which provided criteria to evaluate the methodological quality of the studies. To accurately identify the relationship between the mortality from OHCA and smoking, the heterogeneity and sensitivity of the results were analysed in this study.

## Conclusion

This is the first systematic review and meta-analysis on the association between cigarette smoking and mortality in patients with OHCA. The results would suggest that smokers had lower mortality than non-smokers. However, due to a lack of data, the ‘smoker’s paradox’ is not a definitive concept, and other covariate effects require further consideration and analysis. Efforts are needed to initiate large multi-centre, randomised, prospective clinical trials to evaluate the association between smoking and mortality after OHCAs. In addition, attention should be given to the research on ischemic preconditioning to promote the diagnosis and treatment of OHCAs.
